# Stimulated Salivary Flow Rate, Salivary pH, Viscosity, and Salivary Buffer Capacity and Their Relationship With Molar Incisor Hypomineralization in Mexican Schoolchildren

**DOI:** 10.1155/bmri/1648105

**Published:** 2026-03-23

**Authors:** Alvaro García Pérez, Carmen Seseña Pascasio, Jacqueline Adelina Rodríguez Chávez, María de los Ángeles Martínez Antonio, Karla Lizbeth Murillo Santos

**Affiliations:** ^1^ Laboratory of Public Health Research, Faculty of Higher Studies (FES), Iztacala, National Autonomous University of Mexico (UNAM), Tlalnepantla, State of Mexico, Mexico, unam.mx; ^2^ Specialization in Pediatric Dentistry, Faculty of Higher Studies (FES), Iztacala, National Autonomous University of Mexico (UNAM), Naucalpan of Juarez, State of Mexico, Mexico, unam.mx; ^3^ Department of Comprehensive Dental Clinics, University Center for Health Sciences, University of Guadalajara, Guadalajara, Jalisco, Mexico, udg.mx

**Keywords:** buffer capacity, children, molar incisor hypomineralization, pH, saliva, stimulated salivary flow rate, viscosity

## Abstract

**Aim:**

The aim of this study is to examine the prevalence of molar incisor hypomineralization (MIH), as well as the relationship between stimulated salivary flow rate, salivary pH, viscosity, and salivary buffer capacity with the presence of MIH in 6‐ to 12‐year‐old Mexican children.

**Material and Methods:**

Cross‐sectional study conducted on 296 6‐ to 12‐year‐old schoolchildren selected from a public primary school. Saliva samples were collected and analyzed for resting salivary flow rate, salivary pH, stimulated salivary flow rate, viscosity, and salivary buffer capacity, using the Saliva‐Check BUFFER kit. MIH was evaluated using the European Academy of Paediatric Dentistry criteria (EAPD).

**Results:**

The prevalence of MIH was found to be 27.7%, whereas 42.9% of participants presented poor oral hygiene. Only four salivary parameters were observed to be associated with the presence of MIH. The probability of the occurrence of MIH was significantly higher in children presenting a high level of salivary viscosity (OR = 2.39; *p* = 0.007), a moderately acidic salivary pH (OR = 2.98; *p* = 0.001), a low/very low stimulated salivary flow rate (OR = 2.49; *p* = 0.006), and a low/very low salivary buffer capacity (OR = 4.24; *p* < 0.001).

**Conclusions:**

A relationship was found between stimulated salivary flow rate, salivary pH, viscosity, and salivary buffer capacity and the presence of MIH in Mexican children. Specific strategies and interventions using remineralizing agents are required to improve both salivary health and the remineralization of teeth damaged by MIH in the child population.

## 1. Introduction

Developmental defects of enamel (DDE) are alterations in the formation of the matrix and the mineralization of the hard tissues of the teeth and mainly present as enamel hypoplasia, molar incisor hypomineralization (MIH), amelogenesis imperfecta, and dental fluorosis [1]. In the last two decades, MIH has become a more significant oral health issue on a global level. Defined as a defect in the mineralization of the enamel characterized by marked white/cream or yellow/brown opacities [2], MIH mainly affects one to four first permanent molars and possibly the permanent incisors and the second primary molars [3]. It has even been observed to damage the second permanent molars [4]. The appearance of MIH lesions may be a consequence of diverse factors related to the prenatal, perinatal, and postnatal stages of life [5]. However, these defects are caused by not only environmental factors but also genetic disorders [5]. MIH children may present enamel structure loss caused by masticatory forces and may develop dental caries. Children with MIH frequently present pain and hypersensitivity, specifically when consuming cold or hot foodstuffs and during toothbrushing, causing a negative impact on the quality of life aspects related to oral health [6]. The reported prevalence of MIH in the child and adolescent population ranges from 5% to 40% [7–9]. A 2008 study conducted in a community in Mexico found that 20.3% of its 6‐ to 8‐year‐old children presented signs of MIH, whereas by 2017, this prevalence had increased to 31.9% [10].

The role played by saliva in MIH remains unclear. An element, which is essential to oral health and surrounds the hard and soft tissue of the oral cavity, saliva comprises organic and inorganic components that reflect the general health of the body [11]. Saliva has also been reported to play a fundamental role in various functions, such as viscosity, the regulation of pH, tooth remineralization, the speed of salivary flow, salivary buffer capacity, microbial activity, and tissue repair [12]. However, saliva is not only able to mitigate the low pH caused by acid exposure but also to act as a transporter of essential ions, such as calcium and phosphate, which are essential for promoting remineralization and detaining demineralization [13].

Saliva has also been found to be a factor influencing the presentation of caries, where, for example, its physicochemical properties, such as salivary flow rate, pH, buffer capacity, and viscosity, have been associated with caries and, moreover, act as markers of caries activity [14]. Furthermore, in children with active caries, saliva has been associated with reductions in the speed of salivary flow, salivary volume, salivary pH, and salivary buffer capacity [15].

The literature reports few studies evaluating salivary parameters in teeth affected by MIH and which have reported contradictory results. For example, Ghanim et al. have reported that the saliva obtained from MIH patients presents altered physicochemical properties, corresponding to low salivary flow rates, moderately viscous saliva, and a low salivary pH [16]. However, Ismayilova et al. did not observe statistically significant differences in salivary pH, stimulated salivary flow, and salivary buffer capacity, solely observing differences in calcium concentration between children with and without MIH [17]. Finally, the salivary analysis conducted by Horta et al. revealed higher calcium and lower phosphate levels in teeth with MIH [18].

Saliva plays a significant protective role against the effects of MIH, mainly via remineralization and acid neutralization. Although in itself, MIH is an enamel development defect, the properties of the saliva may also affect enamel integrity. Therefore, evaluating saliva components would help in the development of preventative strategies aiming to protect the structure of enamel damaged by MIH. Therefore, the objective of the present study was to examine the prevalence of MIH and the relationship between stimulated salivary flow, salivary pH, viscosity, salivary buffer capacity, and the presence of the disorder in Mexican children aged 6–12 years.

## 2. Material and Methods

### 2.1. Ethics Committee Approval

The research protocol was approved by the Ethics Committee of the Iztacala Faculty of Higher Studies at the National Autonomous University of Mexico (CE/FESI/062024/1764), whereas the study was carried out in accordance with the Declaration of Helsinki. Permission from both the leadership team at the public primary school and the children′s parents/guardians was also obtained, while the parents/guardians also provided their signed informed consent. Similarly, in order to participate in the study, the children were required to provide their signed informed assent.

The present study was carried out in adherence with the guidelines set out by the Strengthening the Reporting of Observational Studies in Epidemiology (STROBE) statement.

### 2.2. Study Design

The present cross‐sectional study was conducted from February to July, 2025, using data obtained from a public primary school located in a community in Naucalpan de Juárez, in the State of Mexico. The *inclusion criteria* were as follows: schoolchildren aged 6–12 years old, regardless of their gender; presenting one to four permanent upper and lower incisors; presenting one to four completely erupted first permanent molars; and presenting authorization to participate in the study. The *exclusion criteria* were as follows: having lived in another place of residence for more than 6 months during the first 6 years of life; presenting any type of orthodontic apparatus that would interfere with the examination of the tooth surface; failing to cooperate during the oral examination; and presenting other forms of enamel damage. A total of 400 informed consent forms were distributed to the schoolchildren, of which 350 were signed by parents/guardians, corresponding to a response rate of 87.5%. Based on the exclusion criteria, 54 children did not participate in the present study, leaving a total of 296 participants.

### 2.3. Study Size

The sample size was calculated using the formula for a single proportion. Assuming that 20% of the subjects in the population presented the factor of interest (MIH), the study would require a sample size of 246 for estimating the expected proportion with 5% absolute precision and 95% confidence [19].

### 2.4. Clinical Oral Examination

The clinical oral examination was conducted in the public school selected by two dentists using dental mirrors #5 (Hu Friedy, Chicago, United States) and a WHO probe (Hu Friedy, Chicago, United States). The examination of the schoolchildren′s oral cavity adhered to the corresponding infection control standards and was conducted by two examiners who participated in both the training and calibration exercises, which consisted of two steps (theoretical and clinical) that used the MIH and OHI‐S indexes. The interexaminer and intraexaminer agreement for MIH and OHI‐S corresponded to respective Cohen′s kappa coefficients of > 82% and > 0.85%.

### 2.5. Dependent Variable: MIH

The MIH evaluation comprised an examination of the vestibular, occlusal/incisal, and palatal surfaces of all the permanent erupted molars and incisors, classifying them under the EAPD criteria [20]. The participant was classified as presenting MIH when one of their incisors or first permanent molars presented signs of MIH, with the result then dichotomized into absence/presence.

### 2.6. Covariates

The variables considered by the present study were age in years and sex (boy/girl), whereas oral hygiene was evaluated using the Simplified Oral Hygiene Index (OHI‐S), which comprises debris and calculus scores obtained for selected tooth surfaces. The buccal and lingual surfaces of six permanent teeth (identified using the index) were examined, with oral hygiene, as assessed prior to the MIH examination, classified as either poor (OHI‐S score ≥ 2) or good (OHI‐S score < 2) [21].

### 2.7. Measurement of Saliva Variables

The saliva samples were evaluated using the Saliva‐Check BUFFER kit (GC, Tokyo, Japan), which evaluates resting salivary flow rate, viscosity, salivary pH, stimulated salivary flow rate, and salivary buffer capacity, following the manufacturer′s instructions for collection and analysis procedures. Table 1 presents the measurement scales for each of the saliva evaluations conducted by the present study.

**Table 1:** Diagnostic criteria for saliva simples (Saliva‐Check BUFFER) in Mexican schoolchildren.

Salivary variable	Scale		
*Resting saliva*			
Flow rate	< 60 seg: flow normal	> 60 seg: flow low	
Viscosity	Normal (watery‐clear)	High (frothy‐bubbly)	High (frothy‐sticky)
Acidity (salival pH)	Healthy saliva (green/pH = 6.8–7.8)	Moderately acidic (yellow/pH = 6.0–6.6)	Highly acidic (red/pH = 5.0–5.8)
*Stimulated saliva*			
Flow rate/5 min	Normal (> 5.0 mL)	Low (3.5–5.0 mL)	Very low (< 3.5 mL)
Buffering capacity	10–12 Normal (green)	6–9 Low (yellow)	0–5 Very low (red)

All of the saliva evaluations were carried out at the public primary school of interest and the data obtained then analyzed on‐site immediately after collection. The child was asked to relax and sit upright, under natural light, in a classroom specifically selected for sampling. The children were instructed not to consume food or beverages, brush their teeth, or rinse their mouth for the 1 h prior to sampling, which was conducted between 8:00 and 10:00 am to reduce the potential influence of circadian variations on the salivary flow measurement. The average sample collection time was approximately 10 min.

### 2.8. Statistical Analysis

The statistical analysis was carried out using the Stata 19 software (Stata Corp, College Station, Texas, United States). First, a descriptive analysis of the population was performed. Bivariate analysis was then undertaken to establish the relationship between the salivary parameters, sex, and age in the schoolchildren both with and without MIH, using the Pearson chi‐squared test for categorical variables and the Mann–Whitney *U* test for continuous variables. The association between the salivary parameters (resting flow rate, viscosity, pH, stimulated flow rate, and buffering capacity) and MIH was evaluated by the application of a logistic regression model adjusted for sex, whereas the odds ratio (OR) was calculated with 95% confidence intervals (95% CI). Model diagnostic tests were conducted using the Hosmer–Lemeshow goodness of fit test, with values of *p* < 0.05 considered statistically significant.

## 3. Results

**Figure 1:**
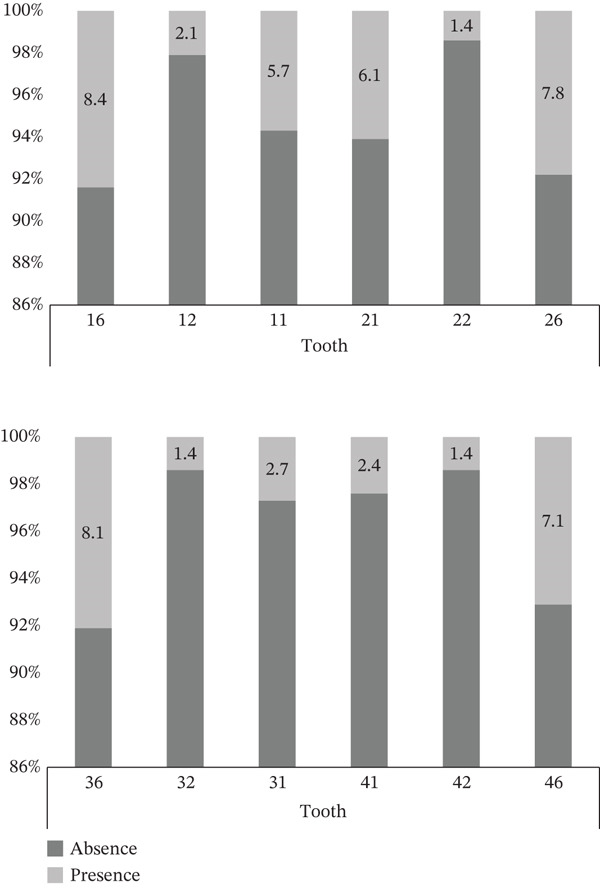
Percentage distribution of teeth affected by molar incisor hypomineralization (MIH) in Mexican schoolchildren.

The present study examined a total of 296 6‐ to 12‐year‐old schoolchildren, the average age of whom was 9.07 (±1.8) years (9.1 [±1.8] boys and 9.0 [±1.7] girls; *p* = 0.693] and of whom 50.3% were boys. The prevalence of MIH was 27.7% (43.9% for boys and 56.1% for girls, *p* = 0.170), whereas 42.9% presented poor oral hygiene. Figure 1 shows the percentage distribution of teeth with MIH damage, from which it can be observed that the first permanent molars are the most damaged, followed by the upper and lower central incisors.

The children with MIH presented a high resting salivary viscosity (*p* < 0.001), as did those children with a low resting salivary flow (*p* = 0.012). Those children with MIH presented a stimulated salivary flow (*p* < 0.001) that was low/very low in comparison with the normal stimulated salivary flow levels observed. Finally, the children with MIH presented a salivary buffer capacity that was low/very low in comparison with the normal buffer capacities observed (*p* < 0.001) (Table 2).

**Table 2:** Comparison between groups with and without molar incisor hypomineralization (MIH) according to sex, age, and salivary parameters in Mexican schoolchildren aged 6–12 years.

Variables	Non‐MIH‐affected children *n*(%)	MIH‐affected children *n*(%)	*p* ^∗^
Sex			
Boys	113 (52.8)	36 (43.9)	0.170
Girls	101 (47.2)	46 (56.1)	
Age (mean SD)	8.98 (±1.83)	9.42 (±1.66)	0.054 ^∗∗^
Resting saliva flow rate			
< 60 seg: flow normal	208 (97.2)	74 (90.2)	0.012
> 60 seg: flow low	6 (2.8)	8 (9.8)	
Resting saliva viscosity			
Normal	168 (78.5)	42 (51.2)	< 0.001
High	46 (21.5)	40 (48.8)	
Resting saliva pH			
Healthy	138 (64.5)	46 (56.1)	0.183
Moderately acidic	76 (35.5)	36 (43.9)	
Highly acidic	0 (0.0)	0 (0.0)	
Stimulated saliva flow rate			
Normal	145 (67.8)	26 (31.7)	< 0.001
Very low–low	69 (32.2)	56 (68.3)	
Buffering capacity			
Normal	122 (57.0)	13 (15.8)	< 0.001
Very low–low	92 (43.0)	69 (84.2)	

^∗^Chi‐squared test.

^∗∗^Mann–Whitney *U* test.

### 3.1. Multivariate Analysis

To evaluate the association between the salivary parameters and MIH, a logistic regression analysis was undertaken, using the resting flow rate, viscosity, pH, stimulated flow rate, buffer capacity, and sex as predictors (Table 3). Only four salivary parameters were found to be associated with the presence of MIH. The probability of MIH occurring in the participants was significantly higher for those presenting a high level of salivary viscosity (OR = 2.39 [95% CI 1.26–4.52]; *p* = 0.007), a moderately acidic salivary pH (OR = 2.98 [95% CI 1.55–5.73]; *p* = 0.001), low/very low stimulated salivary flow (OR = 2.49 [95% CI 1.29–4.81]; *p* = 0.006), and a low/very low salivary buffer capacity (OR = 4.24 [95% CI 2.04–8.83]; *p* < 0.001). Lastly, the value of *p* = 0.312 observed in the Hosmer–Lemeshow goodness‐of‐fit test applied indicates that the model used by the present study was a good fit for the data obtained.

**Table 3:** Adjusted odds ratios from the logistic regression model between salivary parameters and molar incisor hypomineralization (MIH) in Mexican schoolchildren.

Variables	Crude odds ratio (95% CI)^a^	p	Adjust odds ratio (95% CI)^a^	*p*
Sex				
Boys	*Reference*	*—*	*Reference*	—
Girls	1.42 (0.87–2.32)	0.153	1.16 (0.65–2.08)	0.611
Resting saliva flow rate				
Flow normal	*Reference*	*—*	*Reference*	—
Flow low	3.74 (1.25–11.1)	0.018	2.36 (0.72–7.70)	0.152
Resting saliva viscosity				
Normal	*Reference*	*—*	*Reference*	—
High	3.47 (2.02–5.98)	< 0.001	2.39 (1.26–4.52)	0.007
Resting saliva pH				
Healthy	*Reference*	*—*	*Reference*	—
Moderately acidic	1.42 (0.84–2.38)	0.184	2.98 (1.55–5.73)	0.001
Stimulated saliva flow rate				
Normal	*Reference*	*—*	*Reference*	—
Very low–low	4.52 (2.62–7.81)	< 0.001	2.49 (1.29–4.81)	0.006
Buffering capacity				
Normal	*Reference*	*—*	*Reference*	—
Very low–low	7.03 (3.66–13.5)	< 0.001	4.24 (2.04–8.83)	< 0.001

Abbreviations: CI, confidence interval; OR, odds ratio.

^a^Log likelihood = −139.92095, Hosmer–Lemeshow = 0.3121.

## 4. Discussion

The present study found that high salivary viscosity, a moderately acidic salivary pH, a low/very low stimulated salivary flow, and a low/very low salivary buffer capacity were associated with the presence of MIH in 6‐ to 12‐year‐old schoolchildren.

Although the literature reports a relationship between salivary parameters and the presence of caries in children [22, 23], few studies have explored the relationship between salivary parameters and MIH. For example, Ghanim et al. found that saliva taken from patients with MIH showed altered physicochemical properties, such as low salivary flow rates, moderately viscous saliva, and a low salivary pH [16]. However, Ismayilova et al. did not find statistically significant differences in terms of the salivary pH, stimulated salivary flow rate, and salivary buffer capacity [17]. One possible explanation for these findings is that the more marked changes in the physicochemical properties of the saliva of children with MIH may result from a medical condition [16]. For example, children with stunting have been found to present altered salivary parameters, including reduced salivary flow, acidic salivary pH, a higher salivary viscosity, and a lower salivary buffer capacity, suggesting a deterioration in salivary function [24]. Moreover, children with celiac disease, enamel defects, and caries have been observed to present a very low salivary flow, although no changes to salivary buffer capacity were observed by the corresponding study [25]. The composition of the saliva shows a significant variation in younger people, changing with age as the salivary glands mature [26]. In preschool and primary school age groups, the unstimulated salivary flow rate increases with age [27]. Therefore, ascertaining the physicochemical properties of the saliva of the child and adolescent populations presenting MIH is a research priority, whereas combined research is also required on the clinical characteristics of MIH and the corresponding etiological and technical factors, via a method such as an analysis of the salivary proteome.

As a clinical metric, salivary proteomics specifically involves the application of proteomic techniques to study the complete range of proteins present in the saliva and associated with oral and systemic diseases [28]. Considering that changes to the physicochemical properties of the saliva have been associated with oral pathologies, it is possible that children presenting MIH may also present an altered salivary proteome. Bekes et al. examined the salivary protein composition in children with MIH and aged 6–14 years, finding that, of the 618 salivary proteins identified with high confidence, 88 were observed exclusively in those patients with MIH [29]. Moreover, Pappa et al. identified 142 proteins with differential expression in children with MIH [30]. Both the foregoing studies concluded that the protein composition of saliva is impacted in patients with MIH, indicating a catabolic environment linked to inflammation. Further research is required to evaluate the interaction between changes to the salivary proteome and the etiological factors associated with the presence of MIH.

Salivary viscosity is another parameter influencing the protective functions of saliva. The present study found that children with MIH are more likely to present a high level of salivary viscosity (OR = 2.39; *p* = 0.007). Increased viscosity can compromise both the lubricating function of the saliva, thus reducing the self‐cleaning efficacy of the oral cavity, and the level of protection provided to the oral mucosa and the teeth against enamel erosion, bacterial infection, and halitosis [24, 31]. Furthermore, a high level of viscosity can restrict the ingestion of food [24].

The present study found a lower salivary buffer capacity (*OR* = 4.24; *p* < 0.001) and stimulated salivary flow (OR = 2.49; *p* = 0.006) in children with MIH than in the healthy children examined. A significant factor helping to regulate salivary pH, improving tooth remineralization, and preventing the growth of aciduric bacteria [32], salivary buffer capacity is altered by metabolic and hormonal changes, as well as by the individual′s health in general [33]. Studies have reported that salivary buffer capacity depends on the bicarbonate, phosphate, and protein concentration and has even been associated with salivary flow [34]. In cases of MIH in which the enamel is porous and weak and presents both a lower quality and quantity of minerals (Ca, P, and Cl) and a higher protein content [35], the protective function of saliva is of even more critical importance. A reduction in salivary flow or a change to its composition due to certain medical conditions or medication increases the risk of caries lesions and the loss of tooth structure in children with MIH. Therefore, research is required to explore the influence of salivary buffer capacity on the enamel damaged by MIH, given that the majority of the studies conducted in this area have focused on the effects of salivary buffer capacity on teeth damaged by caries.

The present study found a moderately acidic salivary pH (OR = 2.98; *p* = 0.001) in children with MIH. The combined effect of salivary pH and buffer capacity cause pH fluctuations, thus changing the product of the ionic activity in the saliva, particularly those products, such as calcium and phosphate, associated with the demineralization and remineralization processes [36]. For this reason, it can be suggested that the saliva of children with MIH contains low levels of calcium and phosphate and prevents the neutralization of acids, meaning that the surface of the enamel damaged by MIH does not remineralize, thus compromising its integrity. The literature reports the positive effect of remineralizing agents on teeth damaged by MIH, with a consequent increase in the remineralization of the slight and moderate defects caused by MIH observed over a period of 3–12 months [37, 38]. Therefore, the use of remineralizing agents helps to increase calcium and phosphate concentrations in teeth damaged by MIH, thus preventing the appearance or progression of caries lesions.

One of the limitations of the present study was the absence of biochemical analysis that would ascertain the calcium, phosphate, bicarbonate, protein, amylase, and uric acid concentrations and establish the antioxidant capacity of the saliva analyzed. Factors related to the prenatal, perinatal, and postnatal stages of the child′s life were also not evaluated. Nevertheless, the results obtained by the present study do provide valuable information on the salivary parameters of teeth presenting MIH. It is also important to consider that results found cannot be generalized to the child population in general. Longitudinal studies are required to evaluate the interactions among the salivary parameters, developmental defects in the enamel, and the etiological factors associated with the presence of MIH in the child and adolescent population.

## 5. Conclusions

A relationship was found between stimulated salivary flow rate, salivary pH, viscosity, and salivary buffer capacity with the presence of MIH in Mexican children. High levels of salivary viscosity, a moderately acidic salivary pH, and low/very low levels of both salivary flow and salivary buffer capacity were significantly more common in the group of children presenting MIH. The data obtained from the analysis conducted on the saliva may serve as reference metrics for clinical diagnosis, especially as part of the clinical follow‐up, management, and treatment applied for people with MIH. Furthermore, the findings obtained reveal that specific strategies and interventions using remineralizing agents are required to improve both salivary health and the remineralization of teeth damaged by MIH in the child population.

## Funding

This study was supported by Programa de Apoyo a Proyectos de Investigación e Innovación Tecnológica (PAPIIT) UNAM (IN201525).

## Ethics Statement

The study was approved by the Ethics Committee at the Iztacala Faculty of Higher Studies of the National Autonomous University of Mexico (CE/FESI/062024/1764).

## Conflicts of Interest

The authors declare no conflicts of interest.

## Data Availability

The data that support the findings of this study are available from the corresponding author upon reasonable request.
